# Precision Medicine for *BRCA*/*PALB2*-Mutated Pancreatic Cancer and Emerging Strategies to Improve Therapeutic Responses to PARP Inhibition

**DOI:** 10.3390/cancers14040897

**Published:** 2022-02-11

**Authors:** Daniel R. Principe

**Affiliations:** Medical Scientist Training Program, University of Illinois College of Medicine, Chicago, IL 60612, USA; principe@illinois.edu; Tel.: +1-(312)-413-7271

**Keywords:** pancreatic ductal adenocarcinoma, precision medicine, homologous recombination deficiency, PARP inhibitor, *BRCA*, *PALB2*

## Abstract

**Simple Summary:**

For the small subset of pancreatic ductal adenocarcinoma (PDAC) patients with loss-of-function mutations to *BRCA1/2* or *PALB2*, both first-line and maintenance therapy differs significantly. These mutations confer a loss of double-strand break DNA homologous recombination (HR), substantially altering drug sensitivities. In this review, we discuss the current treatment guidelines for PDAC tumors deficient in HR, as well as newly emerging strategies to improve drug responses in this population. We also highlight additional patient populations in which these strategies may also be effective, and novel strategies aiming to confer similar drug sensitivity to tumors proficient in HR repair.

**Abstract:**

Pancreatic cancer is projected to become the second leading cause of cancer-related death by 2030. As patients typically present with advanced disease and show poor responses to broad-spectrum chemotherapy, overall survival remains a dismal 10%. This underscores an urgent clinical need to identify new therapeutic approaches for PDAC patients. Precision medicine is now the standard of care for several difficult-to-treat cancer histologies. Such approaches involve the identification of a clinically actionable molecular feature, which is matched to an appropriate targeted therapy. Selective poly (ADP-ribose) polymerase (PARP) inhibitors such as Niraparib, Olaparib, Talazoparib, Rucaparib, and Veliparib are now approved for several cancers with loss of high-fidelity double-strand break homologous recombination (HR), namely those with deleterious mutations to *BRCA1/2*, *PALB2*, and other functionally related genes. Recent evidence suggests that the presence of such mutations in pancreatic ductal adenocarcinoma (PDAC), the most common and lethal pancreatic cancer histotype, significantly alters drug responses both with respect to first-line chemotherapy and maintenance therapy. In this review, we discuss the current treatment paradigm for PDAC tumors with confirmed deficits in double-strand break HR, as well as emerging strategies to both improve responses to PARP inhibition in HR-deficient PDAC and confer sensitivity to tumors proficient in HR repair.

## 1. Introduction

Pancreatic ductal adenocarcinoma (PDAC) is projected to become the second leading cause of cancer-related death in the United States, with an overall survival rate of 10% [1]. At the present time, nearly all pancreatic cancers are treated similarly with a combination of surgery, if eligible, and broad-spectrum chemotherapy [2]. Though this approach offers a survival benefit to most patients, mounting evidence suggests that there are several genomically distinct PDAC subtypes, many of which may ultimately dictate therapeutic responses [3,4]. While genomic subtyping has yet to influence the current treatment paradigm for most PDAC patients, there is a notable exception for patients harboring a deleterious mutation to either *BRCA1*, *BRCA2*, or *PALB2* [5,6]. Patients with such mutations are thought to have deficiency in high-fidelity double-strand break homologous recombination (HR), limiting the ability of tumor cells to repair double-stranded DNA breaks. In many cancers, mutation to these and other functionally related genes have long been used to predict sensitivity to selective poly (ADP-ribose) polymerase (PARP) inhibitors such as Niraparib, Olaparib, Talazoparib, Rucaparib, and Veliparib [7].

This approach is based on the ability of PARP inhibitors to limit the capacity for single-strand break repair, leading to the accumulation of DNA damage and eventually cell death in the setting of genetic HR deficiency (HRD) [8,9]. Accordingly, PARP inhibition has shown substantial efficacy in the treatment of several *BRCA*-mutated tumors, including breast, ovarian, prostate, and now pancreatic cancers [5,10,11,12]. Additionally, these mutations are associated with differences in chemo-sensitivity, particularly regarding platinum-based alkylating agents (discussed in detail below). Thus, for PDAC tumors with loss-of-function mutations in HR genes *BRCA1*, *BRCA2*, and *PALB2*, both first-line and maintenance therapy differ significantly [2]. Here, we discuss the known roles of HRD in PDAC, with a particular focus on the mechanisms of altered drug responses and the current guidelines for treatment. Additionally, we highlight emerging strategies to further enhance drug sensitivity for patients with HRD, as well as new strategies targeting HR to confer sensitivity to these approaches in HR-proficient tumors.

## 2. PDAC and Genetic Defects in Homologous Recombination

As with many cancers, there is a strong genetic component to PDAC. Recent estimates suggest that as many as 10% of PDAC cases can be attributed to a familial predisposition [13]. Accordingly, patients with a first-degree relative with PDAC have a substantial risk-increase [14,15], particularly in the presence of select environmental risk factors [16]. While any number of predisposing genetic alleles have been described [17], several inherited defects in double-strand break HR have been shown to increase PDAC risk. For example, ataxia-telangiectasia is associated with an increased risk of PDAC [18]. This syndrome involves an inherited loss-of-function mutation to the DNA response and repair gene *ATM*, leading to increased genetic instability due to a loss of high-fidelity double-strand break HR and dysregulation of cell cycle checkpoints [19]. Consistent with the role of ATM as a tumor suppressor gene in PDAC, loss of *Atm* in mice accelerates tumor development, increasing genomic instability and enhancing metastasis [20,21].

Hereditary breast and ovarian cancer (HBOC) syndrome caused by deleterious mutations in *BRCA1* and/or *BRCA2* also confers an increased risk of PDAC, again due to the presumptive loss of double-strand break HR [22]. This is consistent with evidence in mouse models, where germline *Brca2* heterozygosity cooperated with oncogenic KRAS to promote PDAC development [23], with subsequent studies suggesting that *Trp53* signaling must be modified prior to *Brca2* inactivation for *Brca2*-deficient cells to form tumors [24]. Similarly, truncating mutations to *PALB2* increase PDAC risk [25], though less is known regarding *PALB2* in PDAC biology. However, clinical data strongly suggest that patients with a known or family history of these genetic events may benefit from early screening [26,27].

While these mutations are known to predispose for PDAC, it is important to note that the rates of *ATM*, *BRCA1/2*, and *PALB2* mutations are relatively uncommon in PDAC. In the case of *ATM*, the rates are highly varied with somatic mutations identified in 2–18% of PDAC tumors and germline mutations identified in 1–34% [28]. For *BRCA* and *PALB2*, germline mutation frequencies are estimated to be present in 5–9% of patients [29,30,31,32]. When analyzing publicly available datasets of PDAC patients (*N* = 741) as described previously [33,34,35,36], the frequency of these mutations was far lower, with a composite mutation rate of 2.97% for *ATM*, 1.08% for *BRCA1*, 1.48% for *BRCA2*, and 0.54% for *PALB2* (Table 1). While several of these mutations are known to be oncogenic, presumed to confer loss-of-function and support tumorigenesis, several are poorly described and the impact on HR is unknown (Table 2).

Additional genomic studies have offered varying mutation frequencies for genes involved in DNA HR, particularly when including non-classic genes that may be associated with HRD. For example, a study of 60 PDAC patients already tested for *BRCA* mutation were subject to additional genetic testing using a panel with 24 other cancer susceptibility genes. Using this approach, the authors detected eight (13.3%) pathogenic or potentially pathogenic mutations. Four of these mutations were found in non-BRCA genes, with two affecting ATM, one affecting PALB2, and one affecting RAD50 [37]. Consistent with these observations, our recent study using an expanded panel of 22 genes associated with HR, demonstrated an overall mutation frequency of 15% [35], with similar results observed in a study using a more focused molecular panel of six genes [38]. Accordingly, a large-scale meta-analysis evaluated several surrogate markers of HRD in PDAC, focusing on *BRCA1*, *BRCA2*, *PALB2*, *ATM*, *ATR*, *CHEK2*, *RAD51*, and *FANC*. Using this expanded panel, HRD prevalence ranged from 14.5–16.5% using targeted next-generation sequencing, and 24–44% via whole-genome or whole-exome sequencing [39]. Hence, the rate of these and other mutations to the HR repair pathway warrant continued exploration in larger sample sizes with more standardized methodology.

## 3. Chemotherapy for *BRCA* and *PALB2*-MUTATED PDAC

Chemotherapy is the backbone of treatment for both localized and disseminated PDAC [2]. For the majority of patients, first-line treatment consists of FOLFIRINOX (5-Flurouracil, Leucovorin, Irinotecan, and Oxaliplatin) or Gemcitabine with Nab-Paclitaxel [40]. Second-line therapies are far more varied. For example, Gemcitabine with Nab-Paclitaxel can be offered to patients who progress on first-line FOLFIRINOX, and Gemcitabine monotherapy can be offered alone as second-line therapy in patients with a comorbidity profile that prevents the use of more aggressive regimens [40]. The combination of 5-FU/Leucovorin and nanoliposomal (Nal) Irinotecan is also approved for patients who have been previously treated with Gemcitabine-based chemotherapy, and is both safe and well tolerated [41,42].

However, current guidelines support the use of alternate treatment regimens for the 5–9% of PDAC patients with deleterious *BRCA1/2* or *PALB2* mutations. This is based on the longstanding hypothesis that tumors with these and related mutations have a loss of high-fidelity double-strand HR and have improved therapeutic responses to platinum-based chemotherapy [43,44], a phenomenon that has recently been reported in PDAC [45,46]. Specifically, PDAC patients with *BRCA1-*, *BRCA2-,* or *PALB2*-mutated PDAC displayed a 58% response rate to platinum-based chemotherapy compared to 21% of controls [45]. Hence, for PDAC patients with *BRCA1/2-* or *PALB2*-mutated PDAC, front-line therapy varies significantly.

A recent study has demonstrated that patients with borderline resectable, *BRCA*-mutated PDAC are more likely to achieve pathologic complete responses to FOLFIRINOX, which the authors attributed to improved sensitivity to the platinum-agent, Oxaliplatin [47]. Additionally, a recent phase 2 trial has evaluated the combination of Gemcitabine and Cisplatin in *BRCA-* or *PALB2*-mutated PDAC, which led to encouraging 2- and 3-year survival rates of 31% and 18%, respectively [6]. Hence, for PDAC patients with a confirmed mutation in either *BRCA1/2* or *PALB2*, the current treatment guidelines support either FOLFIRINOX or the combination of Gemcitabine and Cisplatin (uptodate.com, accessed on 20 December 2021).

Importantly, the ongoing phase 2 PRIMUS-001 (ISRCTN75002153) and PRIMUS-002 (ISRCTN34129115) trials are also exploring the FOLOX-A regimen (5-Fluorouracil, Leucovorin, Oxaliplatin, and nab-Paclitaxel) in PDAC patients harboring *BRCA1/2* or related mutations. Though results are not yet available, FOLOX-A will be compared to Gemcitabine and nab-Paclitaxel, with a primary endpoint of disease progression during neoadjuvant therapy [48,49]

## 4. PARP Inhibitors and Maintenance Therapy for BRCA-Mutated PDAC

**Figure 1:**
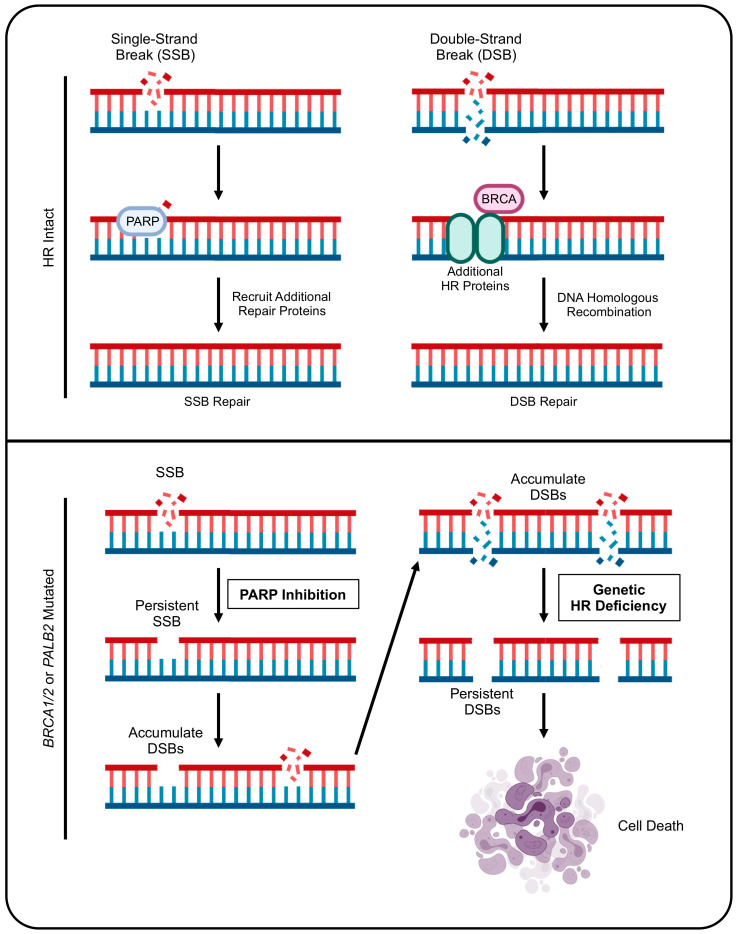
Molecular basis for the efficacy of PARP inhibition in HR-deficient tumor cells. In homologous recombination (HR) proficient PDAC cells, single-strand DNA breaks (SSBs) lead to the rapid synthesis and recruitment of the DNA damage sensor poly (ADP-ribose) polymerase (PARP). In brief, once the PARP enzyme recognizes the DNA breaks via its DNA-binding domain, PARP facilitates base excision repair by acting as a scaffold to recruit additional DNA repair proteins including PNKP, APTX, and LIG3. These additional repair proteins process the SSB, and the gap in the DNA strand is then filled by DNA polymerases and ligated by LIG1. For more severe double-strand breaks (DSBs), protein kinases including ATM and ATR act as damage sensors, driving the recruitment of BRCA proteins to the site of DNA damage. BRCA proteins, assisted by others including BARD1 and BRIP1, organize the assembly of several other DNA repair proteins. This culminates in RAD51 loading, strand invasion, DNA synthesis, and HR-mediated DNA repair to maintain genomic integrity. PDAC cells with deleterious mutations to *BRCA* or *PALB2* are deficient in HR repair, and unable to accommodate DSBs. Therefore, by disrupting the ability of these cells to repair SSB repair using PARP inhibitions, these cells accumulate DSBs, resulting in DNA fragmentation and programmed cell death.

As mentioned, PARP inhibitors are an important part of the treatment for cancers deficient in DNA HR. This is based on longstanding observations that PARP inhibitors impair single-stranded break repair, promoting synthetic lethality in the setting of HRD (Figure 1). Accordingly, PARP inhibitors are now emerging in the treatment of PDAC tumors, namely those with a deleterious *BRCA* mutation [50]. For example, a seminal phase 2 trial evaluated the efficacy of Veliparib in patients with previously treated *BRCA*-mutated PDAC. This study enrolled 16 patients with stage 3/4 disease, 14 of which had received prior platinum-based chemotherapy. Five had a known *BRCA1* mutation, and the remaining 11 had a known *BRCA2* mutation. In this cohort, no confirmed partial responses were observed, four patients showed stable disease, and 11 had progressive disease. Six patients had a grade III toxicity, most commonly fatigue [51].

A subsequent phase 3 trial (POLO) has evaluated the PARP inhibitor Olaparib as maintenance therapy, specifically for *BRCA*-mutated patients who had not progressed during first-line platinum-based chemotherapy. The 154 patients enrolled were randomized into two groups, with 92 receiving Olaparib and 62 a placebo. The Olaparib-treated group demonstrated a significant improvement in progression-free survival (7.8 months compared to 3.8 months on placebo), though an interim analysis did not show a statistically significant increase in overall survival. Olaparib was well tolerated in this study, with no significant difference in health-related quality of life between groups despite the higher rate of grade 3 or worse adverse events in the Olaparib group, most commonly in the form of fatigue, nausea, or other gastrointestinal symptoms [5]. This led to the FDA approval of Olaparib as maintenance therapy for *BRCA*-mutated PDAC in 2019.

The PARP inhibitor Rucaparib has also shown promise for *BRCA/PALB2*-mutated PDAC. An ongoing phase 2 study is exploring single-agent Rucaparib in patients with germline or somatic *BRCA1-, BRCA2-,* or *PALB2*-mutated PDAC that received at least 16 weeks of platinum-based chemotherapy without evidence of chemo-resistance. Of the 42 evaluable patients thus far, the overall response rate was 41.7%, which translated to a median progression-free survival of 13.1 months, and a median overall survival of 23.5 months. Importantly, no new safety concerns were noted [52].

PARP inhibition has also been evaluated in combination with chemotherapy in PDAC patients. For example, a phase 1 study evaluated the combination of Cisplatin, Gemcitabine, and Veliparib in both *BRCA*-mutated and *BRCA*-non-mutated PDAC. This approach was particularly effective in the *BRCA*-mutated group (median overall survival of 22.3 months for BRCA-mutated compared to 11 months for BRCA-non-mutated), leading to a phase 2 study in exclusively *BRCA*-mutated patients [53]. However, in phase 2, Veliparib failed to further improve response rates, and is not currently recommended as a first-line treatment. The combination of Gemcitabine, Cisplatin, and Veliparib was associated with a relatively high rate of serious adverse events, with 48% of patients experiencing neutropenia, 55% thrombocytopenia, and 52% anemia [6]. Additionally, a recent phase 1/2 study has evaluated the combination of Veliparib, 5-Fluorouracil, and Oxaliplatin (NCT01489865) in PDAC patients. The primary endpoint for both phase 2 cohorts of this study was met, and the overall response rate was 26%. The authors noted that this combination had improved therapeutic efficacy in platinum-naïve patients, as well as those with documented mutations in HR, e.g., *BRCA1/2* or *PALB2*. In patients with HRD, the overall response rate was 57% [54].

Several ongoing trials are also exploring PARP inhibitors in PDAC patients, both as monotherapy and in combination with other treatments (summarized in Table 3). Importantly, these include trials exploring: PARP and immune checkpoint inhibitors (NCT05093231, NCT04548752, NCT04753879, NCT04666740, NCT03851614, NCT03404960, NCT04493060, NCT04673448, and NCT04409002), PARP inhibitors and chemotherapy (NCT02890355, NCT01585805, NCT00576654, and NCT03337087), PARP and Vascular Endothelial Growth Factor (VEGF) pathway inhibition (NCT04764084, NCT02498613, NCT04764084), and PARP and ATR inhibition (NCT03682289). The mechanistic intersection between PARP inhibitors and these other therapies are discussed in detail in the following section.

## 5. Emerging Strategies to Improve Therapeutic Responses to PARP Inhibition and/or Sensitize HR-Proficient Tumors

Though PARP inhibition has shown substantial promise for *BRCA*- and *PALB2*-mutated PDAC, several novel combination strategies are also under investigation in solid tumors [55]. Though these are still emerging, particularly in PDAC, several are showing early efficacy. These include approaches to not only advance PARP inhibitors in the treatment of tumors proficient in HR, but also to overcome clinical resistance to PARP inhibition in patients with HRD. Select strategies are summarized below.

### 5.1. PARP Inhibitors in Combination with Inhibitors of Additional DNA Repair Proteins

Several such approaches incorporate inhibitors of proteins involved in HR repair, thereby conferring sensitivity to PARP inhibitors. Ataxia Telangiectasia and Rad3-related protein (ATR) is a serine/threonine-protein kinase that acts as a central regulator of cell cycle checkpoints and HR [56]. Mechanistic data suggest that PARP inhibition leads to an increased reliance on *ATR*/*CHK1* checkpoint signaling [57]. Accordingly, several studies support the combination of ATR and PARP inhibition in HR-proficient tumors. In breast cancer cells, the combination of the ATR inhibitor VE-821 and PARP inhibitors synergize to promote cell death independent of HR-proficiency [58], and ATR inhibition via VE-821 overcomes PARP inhibitor resistance in *BRCA*-deficient cells by disrupting rewired HR and fork protection pathways [59].

Consistent with these observations, the ATR inhibitor BAY 1895344 cooperated with Olaparib in in vivo models of breast and prostate cancer [60], as did the ATR inhibitor AZD6738 in several ATM-deficient cancers [61]. This strategy is emerging in a clinical trial using Ceralasertib, with preliminary results in a small multi-cancer cohort of patients with *ATM*-mutated tumors showing early promise. In brief, 25 patients with confirmed HRD or other DNA-repair deficiency received both Ceralasertib and Olaparib. Though only 8.3% of patients demonstrated responses by RECISTv1.1 criteria, 62.5% derived a clinically meaningful benefit from treatment. This approach was well tolerated, with 32% of patients experiencing a grade 3 or worse toxicity in the form of anemia, neutropenia, or thrombocytopenia [62].

In HR-proficient PDAC, preclinical data support the combination of Olaparib, the ATR inhibitor VE-822, and the dual mTOR kinase/DNA-PK inhibitor CC-115 as maintenance therapy after platinum-based chemotherapy [63]. This approach showed improved survival in orthotopic xenografts when compared to continuous FOLFIRINOX treatment, hallmarked by increased DNA damage and reduced metastasis [63]. Similar strategies are also showing early efficacy in solid tumors. For example, several inhibitors of cell cycle regulators are under investigation in combination with PARP inhibitors in both HR-deficient and HR-proficient tumors. Additionally, CDK12 inhibition via Dinaciclib has been shown to sensitize HR-proficient, *BRCA*-wild type triple negative breast cancer (TNBC) cells to PARP inhibitors, overcoming both acquired and de novo drug resistance [64]. Similar results were observed using CDK2 inhibition in *BRCA1*-mutated breast cancer [65]. Recently, the CDC7 inhibitor TAK-931 has also been shown to improve responses to PARP inhibition in several tumor types, as well as sensitize tumor cells to DNA-damaging chemotherapy [66].

Other targets have also been suggested, notably DNA polymerase Polθ (Polθ). Pharmacologic inhibition of Polθ via ART558 induced DNA damage and synthetic lethality in BRCA-mutated tumor cells, and synergized with PARP inhibition [67]. Though not directly a DNA repair protein, therapeutic inhibition of the nucleotide salvage protein DNPH1 also improved responses to PARP-inhibition in *BRCA*-mutated tumor cells, and was able to overcome acquired resistance to PARP inhibition when combined with the cytotoxic nucleotide 5-hydroxymethyl-deoxyuridine [68]. Similar results were observed using the dual WEE1 and Polo-like kinase 1 (PLK1) inhibitor AZD1775 in gastric cancer cells, which disrupted HR repair and the DNA damage checkpoint, as well as sensitized HR-proficient cells to Olaparib [69]. In high-grade serous ovarian cancer, the CHK1 inhibitor Prexasertib also conferred increased sensitivity to PARP inhibition independent of HR proficiency, compromising both HR repair and replication fork stability [70]. Hence, as these and other strategies directly targeting DNA repair pathways continue to show promise, select combinations warrant continued investigation in PDAC.

### 5.2. PARP and Epigenetic Inhibitors

In addition to strategies targeting DNA repair processes directly, a number of epigenetic inhibitors are also being explored with PARP inhibitors in cancer, including Bromodomain and Extra-Terminal motif (BET) inhibitors. BET proteins include BRD2, BRD3, BRD4, and BRDT [71,72,73]. These proteins recognize acetylated lysine residues via their bromodomains, directing several cellular processes ranging from chromatin remodeling to transcriptional co-activation [74,75,76]. BET proteins have several important roles in PDAC pathobiology [77], and BET inhibitors have shown early promise in preclinical models [78,79]. Several emerging studies suggest an important role for BET proteins as an upstream regulator of double-strand break HR [80,81,82]. Consistent with these observations, a drug synergy screen identified BET inhibition via JQ-1 as an effective means to sensitize HR-proficient breast, ovarian, and prostate cancer cells to Olaparib, in part through impaired translation of *BRCA1* and *RAD51* [83]. Similar results were observed using the BET inhibitor INCB054329 in ovarian cancer cells [84], further supporting the potential of combined BET and PARP inhibition in solid tumors. As emerging data also suggest that BET inhibitors can cooperate with immune checkpoint inhibition in PDAC [36,85], the combination of BET, PARP, and immune checkpoint inhibitors also warrants consideration.

In addition to BET inhibitors, other epigenetic inhibitors are also showing early promise, many of which directly target posttranslational histone modification. For instance, a therapeutic inhibition of Histone Deacetylases (HDACs) is emerging as an additional means of disrupting homologous recombination, largely by reducing the expression of key HR genes such as RAD51 [86,87,88,89]. Based on these findings, several preclinical studies have evaluated the combination of HDAC and PARP inhibition. In TNBC cells, the HDAC inhibitors Suberoylanilide hydroxamic acid (SAHA) and Belinostat cooperated with Olaparib, particularly in cells harboring a deleterious *BRCA1* mutation [90]. Similar findings were observed in HR-proficient TNBC cells, in which HDAC inhibition improved responses to Veliparib irrespective of BRCA1 status [91]. As studies in several tumor types now support the concept of HDAC-inhibitors mimicking a *BRCA*-mutated phenotype and enhancing the tumoricidal effects of PARP inhibitors [92,93,94], this concept warrants exploration in PDAC patients, particularly as the FDA-approved HDAC inhibitor Panobinostat has shown similar results in preclinical testing [95].

In addition to HDAC inhibitors, select inhibitors of DNA methyltransferases (DNMTs) are also showing potential as a means of improving responses to PARP inhibition. A seminal study explored combined DNMT and PARP inhibition in acute myeloid leukemia (AML) and *BRCA*-wild type breast cancer cells, showing synergistic tumor cytotoxicity [96]. In ovarian cancer cells, the DNMT inhibitor Guadecitabine synergized with Talazoparib independent of *BRCA*-status [97]. Similarly, in non-small cell lung cancer cells, DNMT inhibitors induced a phenotype that mimicked a deleterious *BRCA* mutation, and increased sensitivity to both PARP inhibition and ionizing radiation [98]. Hence, though epigenetic inhibitors are still emerging in PDAC [99], this is another potentially useful strategy to improve clinical responses to PARP inhibition or expand their use into HR-proficient tumors.

### 5.3. Additional Combination Strategies

Several additional combination strategies are also showing promise. As mentioned, recent studies have explored the combination of VEGF and PARP inhibitors. Following a promising phase 1 trial [100], a landmark phase 2 trial evaluated the addition of the VEGF signaling inhibitor Cediranib to Olaparib in women with recurrent platinum-sensitive, high-grade serous or endometrioid ovarian cancer. This approach showed a significant improvement in progression-free survival, particularly for patients lacking a deleterious *BRCA* mutation. However, 27.3% of patients in the combination arm experienced a grade 3 or worse adverse event, most commonly diarrhea or hypertension. Hence, the authors recommended that investigations should include assessments of quality of life due to the side-effect profile. [101,102]. Similar combinations are showing preclinical promise in other tumor types, including *KRAS*-mutated colorectal cancer cells [103], though this has yet to be evaluated in PDAC.

Other proposed targets include Aldehyde dehydrogenase 1 family, member A1 (ALDH1A1), which has very recently been linked to microhomology-mediated end joining (MMEJ) and resistance to PARP inhibition. In brief, in ovarian cancer cells, Olaparib increased ALDH1A1 expression through the BET protein BRD4, thereby activating alternate DNA repair pathways in cells harboring a *BRCA2* mutation. Accordingly, the ALDH1A1 inhibitor NCT-501 synergized with Olaparib in cell culture and xenograft models of *BRCA2*-mutated ovarian cancer [104]. Similar results were observed using combined ALDH and ATM/ATR inhibitors in HR-proficient ovarian cancer cells, substantiating ALDH1A1 and related enzymes as a potential target for therapy [105].

Several studies are also exploring the effects of the PI3K pathway on responses to PARP inhibitors. The intersection between PI3K signaling and HR has long been of interest, as PTEN-deficient tumors have been suggested to have reduced expression of RAD51, thereby conferring improved sensitivity to PARP inhibition. Though encouraging, this is rather controversial and PTEN status is not generally considered a useful predictor for PARP inhibitor sensitivity at this time [106,107,108,109]. Additionally, other studies support therapeutic inhibition of PI3K signaling to improve responses to PARP inhibitors. For example, the pan-PI3K inhibitor BKM120 improved responses to Rucaparib by suppressing HR in glioblastoma cells [110]. In *PIK3CA*-mutated ovarian cancer cells, BKM120 cooperated with Olaparib, in part through downregulation of *BRCA1* [111], with similar results observed in *PIK3CA*-wild type cells [112]. In HR-proficient TNBC cells, inhibition of the PI3K target mTOR enhanced the responses to Talazoparib by suppressing HR repair [113]. In early phase clinical testing, the combination of Olaparib and the AKT inhibitor Capivasertib is showing early efficacy in recurrent endometrial, TNBC, and ovarian cancers. Of the 31 evaluable patients at the time of the initial report, 19% had partial responses and 22% stable disease. No serious adverse events were noted [114]. Hence, while this approach is no doubt promising, the role of PI3K signaling in directing HR repair processes and conferring sensitivity to PARP inhibitors is unclear, and should be further explored in PDAC given the driving role of PI3K signaling in PDAC pathobiology [115].

Other potential candidates for therapy have also been proposed including MYC. Selective MYC inhibition has been demonstrated to sensitize TNBC cells and other aggressive MYC-overexpressing tumors to PARP inhibition independent of *BRCA* status [116]. In preclinical models of glioblastoma, MYC-targeted CDK18 has been implicated in resistance to PARP inhibitors by enhancing ATR-mediated HR [117]. As MYC is often overexpressed in PDAC and has central roles in tumor maintenance [118,119], MYC may have utility either as a therapeutic target to improve therapeutic responses or a potential biomarker for PARP inhibitor sensitivity in PDAC tumors.

Several other targets for therapy have been suggested. Notable examples include Glycogen synthase kinase 3β (GSK3β), as a high throughput screen of 99 anti-cancer compounds determined that GSK3β inhibition cooperated with PARP inhibitors in colon cancer, suppressing HR and increasing both replication stress and DNA double-strand breaks [120]. Several studies are also exploring PARP inhibitors combined with inhibitors of receptor tyrosine kinase signaling. In PDAC cells, combined PARP and Fibroblast Growth Factor Receptor 1 (FGFR1) inhibition via PD173074 is synthetic lethal in vitro, showing improved efficacy in tumor xenografts compared to either approach in monotherapy [121]. Similarly, in breast and lung cancers, inhibition of the receptor tyrosine kinase c-MET reduced PARP1 phosphorylation and improved therapeutic responses to PARP inhibitors [122]. Inhibition of downstream signaling has also shown promise, including combined PARP and MEK inhibition, which was highly effective in cancers with an oncogenic RAS mutation [123]. Hence, as PARP inhibitors continue to advance in the treatment of PDAC, these other strategies warrant consideration.

## 6. Summary and Future Direction

There is currently no effective treatment for PDAC. For patients with confirmed HRD in the form of deleterious *BRCA1/2* or *PALB2* mutations, the current treatment paradigm differs significantly from that for other patients, with the combination of Gemcitabine and Cisplatin as an appropriate first-line treatment option [45] and the PARP inhibitor Olaparib as maintenance therapy [5]. Though this approach has shown considerable promise, overall mortality is still high for these patients, underscoring the need to identify new combination strategies to further improve therapeutic outcomes. Importantly, recent evidence suggests that *BRCA-* and *PALB2*-mutated PDAC tumors have distinct molecular characteristics that may pave the way for novel combination strategies that have yet to be evaluated. For example, a recent study evaluated 2818 PDAC specimens using a combination of next-generation sequencing and immunohistochemistry. Though *BRCA* and *PALB2* mutations were relatively uncommon, tumors from *BRCA-* and *PALB2*-mutated patients had distinct mutational profiles compared to non-mutated patients, were more likely to be PD-L1 positive, and had a comparatively high tumor mutational burden (TMB). The authors, therefore, concluded that these data provided rationale to evaluate combined PARP and immune checkpoint inhibition in *BRCA*/*PALB2*-mutated PDAC patients [124]. This approach is now showing early promise in ovarian [125], prostate [126] and lung cancers [127]. As mentioned, this approach is now under evaluation in PDAC, including a phase 2 trial evaluating the combination of Pembrolizumab and Olaparib [128], though this trial and the many similar ongoing studies have yet to post results.

Additionally, there is now consensus that *BRCA* and *PALB2* mutations are not the sole predictors of either HRD or PARP inhibitor sensitivity [129]. For example, in TNBC patients, an unbiased HRD score (an unweighted sum of loss of heterozygosity, telomeric allelic imbalance, and large-scale state transitions) successfully identified several non-*BRCA*-mutated TNBC patients as more likely to respond to platinum chemotherapy [130]. In metastatic prostate cancer, an expanded HRD panel included patients with mutations to *BRCA1*, *BRCA2*, *ATM*, *FANCA*, *CHEK2*, *PALB2*, *NBN*, or *HDAC2*. This identified an HRD frequency of 33%, with 88% of biomarker-positive patients demonstrating therapeutic responses to Olaparib [131]. Similarly, in relapsed platinum-sensitive high-grade ovarian cancer patients, therapeutic responses to Rucaparib were not restricted to *BRCA*-mutated tumors, and were observed in several patients with mutations to other HR genes including *ATM*, *NBN*, *RAD51C*, and *RAD51D* [132]. In PDAC, several studies now suggest that the true rates of HRD in PDAC patients may be underreported using only *BRCA* and *PALB2* mutations as the defining criteria [35,38,39]. Hence, mutations to non-classic HR genes warrant continued exploration as a predictor of drug responses, particularly as PARP inhibitors advance in the treatment of other cancers, often in combination with chemotherapy or radiation [133,134,135,136,137,138,139,140,141,142].

It is also important to note that there is mounting evidence that HR-proficient PDAC patients may also benefit from PARP inhibitors when combined with additional strategies targeting either HR repair processes, both directly and indirectly. This may prove to be an effective strategy to enhance therapeutic responses in PDAC patients, though such approaches have yet to enter clinical evaluation. It is also important to note that clinical responses to PARP inhibitors are not solely driven by HRD. In other cancers, the efficacy of these medications involves several other factors including replication, oxidative, and ER stress [143,144,145,146,147]. Hence, this too warrants investigation in PDAC, particularly given the lack of an effective treatment and high mortality associated with conventional therapeutic approaches.

Finally, while precision medicine can offer significant benefit to select PDAC patients and can even facilitate the testing of at-risk relatives, there are several additional factors that must also be considered. As *BRCA*/*PALB2* mutations are rare in PDAC, this raises the important issue of whether it is justifiable to sequence all tumors given the expense [148]. Though all tumors should be sequenced in theory, in practice this may not be feasible, particularly for the underinsured or patients in low-resource settings. Hence, historically, screening for *BRCA* and similar mutations have been limited to PDAC patients with the relevant family history.

However, cost is seemingly not the only barrier to testing, as *BRCA1/2* testing remains underutilized in cancer patients, even for those with insurance coverage and access to specialty genetic services [149]. The reasons for this are largely unclear, with some hypothesizing that physicians are less likely to order cumbersome and expensive sequencing-based assays when the overwhelming majority of tests will be negative. Though using an expanded HRD panel, as described above, may partially address this problem by potentially identifying additional patients that would benefit from precision medicine approaches, this approach also creates new challenges. Importantly, multi-gene panel testing will likely result in a substantial increase in requests for genetic counselors to interpret variants of uncertain significance, including those affecting genes that are not strongly linked to altered drug responses [150]. Hence, in addition to the important scientific questions regarding precision medicine for PDAC, there are several logistic and socioeconomic questions that also must be addressed.

## Figures and Tables

**Table 1:** Rates of *ATM*, *BRCA1*, *BRCA2*, and *PALB2* mutations in publicly available genomic datasets of PDAC patients compared to other tumor types.

Gene	Data Set	Observed Mutations	Mutation Rate
*ATM*	ICGC Pancreas	1/99	1.01%
	TCGA Pancreas	5/150	3.33%
	QCMG Pancreas	14/383	3.66%
	UTSW Pancreas	2/109	1.83%
	Combined Pancreas	22/741	2.97%
	TCGA Breast	21/977	2.15%
	TCGA Ovarian	4/315	1.27%
*BRCA1*	ICGC Pancreas	0/99	0.00%
	TCGA Pancreas	2/150	1.33%
	QCMG Pancreas	5/383	1.31%
	UTSW Pancreas	1/109	0.92%
	Combined Pancreas	8/741	1.08%
	TCGA Breast	13/977	1.33%
	TCGA Ovarian	12/315	3.81%
*BRCA2*	ICGC Pancreas	0/99	0.00%
	TCGA Pancreas	2/150	1.33%
	QCMG Pancreas	8/383	2.09%
	UTSW Pancreas	1/109	0.92%
	Combined Pancreas	11/741	1.48%
	TCGA Breast	15/977	1.54%
	TCGA Ovarian	11/315	3.49%
*PALB2*	ICGC Pancreas	0/99	0.00%
	TCGA Pancreas	1/150	0.67%
	QCMG Pancreas	2/383	0.52%
	UTSW Pancreas	1/109	0.92%
	Combined Pancreas	4/741	0.54%
	TCGA Breast	7/977	0.72%
	TCGA Ovarian	4/315	1.27%

**Table 2:** Specific mutations to *ATM*, *BRCA1*, *BRCA2*, and *PALB2* mutations in publicly available genomic datasets of PDAC patients.

Gene	Mutation	Mutation Type	OncoKB Analysis	Study
*ATM*	R3008H	Missense	Presumed LOF, Likely Oncogenic	ICGC
	R3008C	Missense	LOF, Oncogenic	QCMG
	R337C	Missense	Presumed LOF, Likely Oncogenic	TCGA
	R3008S	Missense	Presumed LOF, Likely Oncogenic	QCMG
	L1347*	Nonsense	Presumed LOF, Likely Oncogenic	TCGA
	R248*	Nonsense	Presumed LOF, Likely Oncogenic	UTSW
	C1045Lfs*3	FS Insertion	Presumed LOF, Likely Oncogenic	UTSW
	L1347*	Nonsense	Presumed LOF, Likely Oncogenic	QCMG
	X633_splice	Splice	Presumed LOF, Likely Oncogenic	QCMG
	X1726_splice	Splice	Presumed LOF, Likely Oncogenic	QCMG
	A1110Hfs*4	FS Deletion	Presumed LOF, Likely Oncogenic	QCMG
	X2505_splice	Splice	Presumed LOF, Likely Oncogenic	QCMG
	G956Efs*15	FS Deletion	Presumed LOF, Likely Oncogenic	QCMG
	I326Rfs*3	FS Deletion	Presumed LOF, Likely Oncogenic	QCMG
	R2459C	Missense	Unknown	TCGA
	L2780R	Missense	Unknown	TCGA
	P2353H	Missense	Unknown	TCGA
	E2423G	Missense	Unknown	TCGA
	T2934I	Missense	Unknown	TCGA
	R1898Q	Missense	Unknown	TCGA
	F1234S	Missense	Unknown	QCMG
	T939A	Missense	Unknown	QCMG
	L1718V	Missense	Unknown	QCMG
	W2491R	Missense	Unknown	QCMG
	E2444D	Missense	Unknown	QCMG
	V2823F	Missense	Unknown	QCMG
	K387N	Missense	Unknown	QCMG
	L2258P	Missense	Unknown	QCMG
*BRCA1*	X183_splice	Splice	Presumed LOF, Likely Oncogenic	ICGC
	X1778_splice	Splice	Presumed LOF, Likely Oncogenic	ICGC
	A622V	Missense	Unknown	ICGC
	Q687P	Missense	Unknown	ICGC
	T539M	Missense	Unknown	ICGC
	V1590A	Missense	Unknown	TCGA
	A314T	Missense	Unknown	TCGA
	S646G	Missense	Unknown	TCGA
	E515Q	Missense	Unknown	UTSW
*BRCA2*	R3128*	Nonsense	Presumed LOF, Likely Oncogenic	ICGC
	N1784Kfs*3	FS Insertion	Presumed LOF, Likely Oncogenic	ICGC
	X3216_splice	Splice	Presumed LOF, Likely Oncogenic	ICGC
	L2428*	Nonsense	Presumed LOF, Likely Oncogenic	ICGC
	I2296Lfs*10	FS Deletion	Presumed LOF, Likely Oncogenic	ICGC
	E2258K	Missense	Unknown	ICGC
	Q2829H	Missense	Unknown	ICGC
	G1552D	Missense	Unknown	ICGC
	V2716Wfs*17	FS Deletion	Presumed LOF, Likely Oncogenic	TCGA
	S278N	Missense	Unknown	TCGA
	I1017F	Missense	Unknown	TCGA
	T1346N	Missense	Unknown	TCGA
	N1642T	Missense	Unknown	TCGA
	V2079M	Missense	Unknown	TCGA
	P3039S	Missense	Unknown	UTSW
*PALB2*	C768Lfs*82	FS Deletion	Presumed LOF, Likely Oncogenic	ICGC
	A503S	Missense	Unknown	ICGC
	D595A	Missense	Unknown	TCGA
	A308T	Missense	Unknown	TCGA
	W898Efs*29	FS Deletion	Presumed LOF, Likely Oncogenic	UTSW

Abbreviations: Loss-of-function (LOF); frameshift (FS).

**Table 3:** Select ongoing clinical trials exploring PARP inhibitors in pancreatic cancer patients.

PARP Inhibitor	Additional Therapy	NCT Identifier	Phase	Last Status	Notes
Olaparib	-	NCT02184195	3	Active, Not Recruiting	*BRCA*-mutated, non-platinum refractory PDAC
	-	NCT02677038	2	Active, Not Recruiting	PDAC w/“BRCAness” phenotype
	-	NCT04348045	2	Recruiting	PDAC w/“BRCAness” phenotype
	-	NCT04858334	2	Recruiting	*BRCA*- or *PALB2*-mutated PDAC
	-	NCT04005690	1	Recruiting	-
	-	NCT01078662	2	Active, Not Recruiting	*BRCA*-mutated PDAC, Multi-cancer trial
	Pembrolizumab	NCT05093231	2	Announced	PDAC w/High TMB
	Pembrolizumab	NCT04548752	2	Recruiting	*BRCA*-mutated PDAC
	Pembrolizumab	NCT04753879	2	Recruiting	PDAC, after multi-agent, low dose chemotherapy
	Pembrolizumab	NCT04666740	2	Recruiting	HRD and/or highly platinum sensitive PDAC
	Durvalumab	NCT03851614	2	Active, Not Recruiting	Multi-cancer trial
	Ceralasertib	NCT03682289	2	Recruiting	Multi-cancer trial
	Cediranib	NCT02498613	2	Recruiting	Multi-cancer trial
Niraparib	-	NCT03601923	2	Recruiting	*BRCA*-, *PALB2*-, *CHEK2*-, or *ATM*-mutated
	-	NCT03553004	2	Recruiting	-
	-	NCT05169437	2	Announced	*PALB2*-mutated, multi-cancer trial
	Ipilimumab or Nivolumab	NCT03404960	1/2	Recruiting	Platinum-treated PDAC
	Dostarlimab	NCT04493060	2	Recruiting	*BRCA*- or *PALB2*-mutated PDAC
	Dostarlimab	NCT04673448	1	Recruiting	*BRCA*-mutated, multi-cancer trial
	Dostarlimab, Radiation	NCT04409002	2	Active, Not Recruiting	-
	Anlotinib	NCT04764084	1	Announced	PDAC w/confirmed HRD
	PEN-866	NCT03221400	1/2	Recruiting	Multi-cancer trial
Veliparib	5-Fluorouracil, Leucovorin, Irinotecan	NCT02890355	2	Active, Not Recruiting	-
	Gemcitabine, Cisplatin	NCT01585805	2	Active, Not Recruiting	*BRCA*- or *PALB2*-mutated PDAC
	Irinotecan	NCT00576654	1	Active, Not Recruiting	Multi-cancer trial
Rucaparib	-	NCT03140670	2	Active, Not Recruiting	*BRCA*- or *PALB2*-mutated, non-platinum refractory PDAC
	-	NCT04171700	2	Recruiting	HRD, multi-cancer trial
	5-Fluorouracil, Leucovorin, nal-Irinotecan	NCT03337087	1/2	Recruiting	Multi-cancer trial
Talazoparib	-	NCT04550494	2	Recruiting	HRD, multi-cancer trial
		NCT04672460	1	Active, Not Recruiting	*BRCA*-mutated, multi-cancer trial

Abbreviations: Pancreatic ductal adenocarcinoma (PDAC); Tumor mutational burden (TMB); Homologous recombination deficiency (HRD).
